# Muscle-Building Exercise and Weapon Carrying and Physical Fighting Among U.S. Adolescent Boys

**DOI:** 10.1177/08862605221101192

**Published:** 2022-05-12

**Authors:** Kyle T. Ganson, Alexander Testa, Rachel F. Rodgers, Dylan B. Jackson, Jason M. Nagata

**Affiliations:** 1 7938 University of Toronto, Toronto, ON, Canada; 2 12346 University of Texas Health Science Center at Houston, Houston, TX, USA; 3 1848 Northeastern University, Boston, MA, USA; 4 Lapeyronie Hospital, CHRU Montpellier, France; 5 1466 Johns Hopkins Bloomberg School of Public Health, Baltimore, MD, USA; 6 8785 University of California, San Francisco, San Francisco, CA, USA

**Keywords:** muscle-building exercise, weapon carrying, guns, physical fighting, adolescent boys

## Abstract

This study aimed to determine the association between engagement in muscle-building exercise and weapon carrying and physical fighting among adolescent boys. Cross-sectional data from the 2019 Youth Risk Behavior Survey (U.S.) were analyzed (*N* = 4120). Muscle-building exercise was assessed based on the number of days reported in the past 7 days, recategorized into four levels of engagement (no engagement [0 days], low engagement [1–2 days], moderate engagement [3–5 days], and high engagement [6–7 days]). Three forms of weapon carrying (general, on school grounds, gun carrying) and two forms of physical fighting (general, on school grounds) were assessed. Five logistic regression analyses with adjusted odds ratios (AOR) and 95% confidence intervals (CI) were used to determine the association between engagement in muscle-building exercise and weapon carrying and physical fighting, while adjusting for relevant demographic and control variables. Over 75% of participants reported engaging in muscle-building exercise. One in five (19.8%) participants reported any general weapon carrying in the past 30 days, 3.3% reported any weapon carrying at school in the past 30 days, 6.5% reported any gun carrying in the past 12 months, 28.0% reported any general physical fighting in the past 12 months, and 10.7% reported any physical fighting at school in the past 12 months. Logistic regressions showed that, compared to no engagement, participants who reported high engagement of muscle-building exercise had higher odds of general weapon carrying (AOR 2.18, 95% CI 1.54–3.07), gun carrying (AOR 2.12, 95% CI 1.23–3.64), and general physical fighting (AOR 2.07, 95% CI 1.53–2.79). These are novel findings that add to a growing literature related to engagement in muscularity-oriented behaviors among males. Prevention and intervention efforts are needed to ensure that adolescent boys engage in muscle-building exercise in ways that are not harmful and to reduce weapon carrying and physical fighting.

## Introduction

Adolescence is a significant period where psychological, social, and behavioral development occurs, often impacting future health behaviors (Patton et al., 2016; Sawyer et al., 2012). One such health behavior that is often promoted via health and physical education is muscle-building exercise (i.e., weight lifting). Research has underscored the high prevalence of engagement in muscle-building exercise among adolescents. For example, upwards of 20% of adolescent boys, compared to 6% of adolescent girls, report engagement in this form of exercise (Nagata, Ganson, et al., 2020). This form of exercise has many benefits for young people, including injury prevention, particularly in relation to sports participation, bone development, cardiovascular fitness, and improved mental health (Stricker et al., 2020). Given these benefits, it has been recommended by the Academy of Pediatrics (AAP) and the U.S. Department of Health and Human Services that adolescents engage in 2–3 days of muscle-building exercise per week with 1–2 days off per week to ensure physical and psychological recovery (Stricker et al., 2020; U.S. Department of Health and Human Services, 2018). Conversely, despite the benefits, engagement in muscle-building exercise may become problematic if done in an excessive manner outside of the recommended guidance, without proper training and oversight, and influenced by sociocultural body image pressures (Tylka, 2021). Generally, research has investigated the adverse physical effects of high engagement in such exercise, including overtraining and skeletal injury (Stricker et al., 2020). Moreover, prior research has found many negative consequences of engaging in muscle-building behaviors among boys and men. This includes eating disorder behaviors (Ganson, Cunningham, et al., 2022), muscle dysmorphia (Pope et al., 2017), criminal offending (Ganson, Testa, et al., 2021), intimate partner violence (Ganson, Jackson, et al., 2022), and sexual risk behaviors (Ganson, Jackson, et al., 2021), as well as increased likelihood of using anabolic-androgenic steroids that can lead to a plethora of negative physical health effects (e.g., cardiovascular, neuroendocrine, and neuropsychiatric; Nagata et al., 2022; Pope et al., 2014). What remains less well understood is the behavioral correlates of high engagement in muscle-building exercise, such as weapon carrying and physical fighting.

Similar to the engagement in muscle-building exercise, weapon carrying and physical fighting occur overwhelmingly among adolescent boys compared to adolescent girls (de Looze et al., 2019; Jewett et al., 2021; Pham et al., 2017; Pickett et al., 2005; Sattler et al., 2019; Vaughn et al., 2012). From a developmental perspective, these behaviors may occur in relation to the many social and psychological changes that happen during adolescence (Henriksen et al., 2020; Sawyer et al., 2012). For example, regarding weapon carrying, research has shown that adolescents who carry weapons fall into groups characterized by other behavioral risk patterns and profiles that may include physical fighting (Vaughn et al., 2016; Vaughn et al., 2012; Vaughn et al., 2017). Similarly, prior research has underscored that weapon carrying may occur in response to bullying involvement, including both victims and perpetrators, particularly as a means of self-protection (Esselmont, 2014; Oliphant et al., 2019; Pham et al., 2017; Valdebenito et al., 2017; Van Geel et al., 2014). Regarding physical fighting, research has shown similar patterns of risk factors for engagement as with weapon carrying, including bullying victimization and poor grades (Henriksen et al., 2020). In fact, many adolescents endorse fighting in response to being teased or being physically provoked (Farrell et al., 2012). Moreover, physical bulk and muscularity have been shown to be associated with more skilled and successful physical fighting—that is, being in a physical fight without an injury, while injuring the other—among adolescent boys (Beaver et al., 2015). Therefore, a relationship between muscle-building exercise and physical fighting may exist due to those who experience victimization wishing to be more effective at standing up to their aggressors. Relatedly, many undergraduate college-age men report wanting to become more muscular to intimidate other males (79%) and be a better fighter (65%; Frederick et al., 2007), further emphasizing a potential relationship between drive for muscularity and physical fighting. In fact, research has shown that more muscular men perceive themselves to be better fighters; however, this is mediated by one’s level of strength (Muñoz-Reyes et al., 2019). This finding further underscores the potential interconnectedness of muscle-building exercise, as a means to build physical strength, and physical fighting.

These data underscore the complex and problematic nature of weapon carrying and physical fighting, including the behavioral risk factors and correlates of such behaviors. The latter data underscores there may be connections between these risk behaviors and one’s physical body, particularly as it relates to drive for muscularity (Beaver et al., 2015; Frederick et al., 2007; Muñoz-Reyes et al., 2019). A common theoretical thread underpinning these behaviors are the components of hegemonic masculinity, including toughness, strength, and dominance, that boys are often socialized to adhere to and perform through their daily actions (Amin et al., 2018; Connell, 2005). Accordingly, adolescent boys may engage in muscle-building exercise, weapon carrying, and physical fighting as performative attempts to display their adherence to these masculine norms, as has been described previously (Courtenay, 2000; Luciano, 2007). For example, a boy may engage in high levels of muscle-building exercise to gain muscle to appear more physically tough and be a stronger fighter, while also carrying a weapon to assert dominance over others. With this context in mind and given that little research has explored the behavioral correlates of muscle-building exercise, particularly as it relates to the high engagement in such exercise, the aim of this study was to determine the association between engagement in muscle-building exercise and weapon carrying and physical fighting among a diverse and representative sample of U.S. adolescent boys. While no known research has been conducted to investigate these associations, it was hypothesized that adolescent boys who report greater engagement in muscle-building exercise, that is, in excess of the recommended guidance, would be more likely to report weapon carrying and physical fighting as these behaviors will cumulatively display a pattern of risk behaviors and adherence to masculine norms.

## Methods

Data from the 2019 National High School Youth Risk Behavior Survey (YRBS) were analyzed for this study. This analysis was restricted to male participants (*N* = 4120) given the higher prevalence of muscle-building exercise, weapon carrying, and physical fighting compared to females (de Looze et al., 2019; Jewett et al., 2021; Nagata, Ganson, et al., 2020; Pham et al., 2017; Pickett et al., 2005; Sattler et al., 2019; Vaughn et al., 2012). The YRBS is conducted bi-annually by the Centers for Disease Control and Prevention (CDC) to monitor priority health risk behaviors among adolescents. The YRBS utilizes a three-stage cluster sampling method to collect a nationally representative sample of U.S. high school students. The YRBS utilizes active and passive parental consent and is approved by the CDC’s Institutional Review Board (Underwood et al., 2020). This analysis was deemed exempt from further ethics review given that it utilizes publicly available and anonymous data.

### Measures

*Weapon carrying* was assessed using three questions: “During the past 30 days, on how many days did you carry **a weapon** such as a gun, knife, or club?” “During the past 30 days, on how many days did you carry **a weapon** such as a gun, knife, or club **on school property**?” and “**During the past 12** **months**, on how many days did you carry a **gun**? (Do **not** count the days when you carried a gun only for hunting or for a sport, such as target shooting.)” Response options for each question were dichotomized to zero (0) days and one (1) or more days (Ganson & Nagata, 2020; Jewett et al., 2021; Pham et al., 2017; Vaughn et al., 2012).

*Physical fighting* was assessed using two questions: “During the past 12 months, how many times were you in a **physical fight**?” and “During the past 12 months, how many times were you in a **physical fight on school property**?” Response options for each question were dichotomized to zero (0) times and one (1) or more times (Salas-Wright et al., 2017; Sattler et al., 2019).

*Muscle-building exercise* was assessed using the question: “During the past 7 days, on how many days did you do exercises to strengthen or tone your muscles, such as push-ups, sit-ups, or weight lifting?” Responses ranged from 0 to 7 days. Categories were created to capture multiple levels of engagement and to align with recommended physical activity guidance (Stricker et al., 2020). Categories included, no engagement (0 days), low engagement (1–2 days), moderate engagement (3–5 days), and high engagement (6–7 days).

### Demographic and Control Variables

Demographic variables included age, race/ethnicity, and sexual orientation. Control variables included body mass index (BMI) percentile, grades, bullying victimization on school grounds, bullying victimization electronically, any team sports participation, any days of not attending school due to feeling unsafe, and any experience of being threatened or injured with a weapon at school. Body mass index was calculated (BMI = weight/height^2^) and converted into sex and age-specific percentiles in accordance with CDC growth curves and definitions for children under 18 (Centers for Disease Control and Prevention, 2022). These variables were adjusted for given empirical associations with muscle-building behaviors (Nagata, Ganson, et al., 2020; Nagata et al., 2019) and weapon carrying and physical fighting (Ganson & Nagata, 2020; Jewett et al., 2021; Van Geel et al., 2014).

### Statistical Analysis

This analysis was restricted to participants who responded to the muscle-building exercise item, resulting in 2521 missing participants. We tested for differences (chi-square tests) between those included and missing and the weapon carrying and physical fighting variables, as well as age and race/ethnicity. Weapon carrying at school (*p* < 0.001) and physical fighting at school (*p* < 0.001) occurred slightly less frequently among those included. Additionally, those included were slightly older (*p* < 0.001) and more diverse (i.e., non-White; *p* < 0.001).

Descriptive statistics were conducted to characterize the sample. Unadjusted prevalence of weapon carrying and physical fighting by engagement in muscle-building exercise was estimated and differences between groups were determined by the adjusted *F*, a variant of the second-order Rao-Scott adjusted χ^2^. Five logistic regression analyses were conducted to determine the associations between engagement in muscle-building exercise and weapon carrying (general, on school grounds, gun carrying) and physical fighting (general, on school grounds), while adjusting for the demographic and control variables. All analyses included preconstructed nationally representative sample weighting (Underwood et al., 2020) and were conducted in 2021 using Stata 17.

## Results

Among the diverse sample of high school boys, 25.6% were 16 years old, 92% identified as heterosexual, 56% were between the fifth ≤ and <85th BMI percentile (i.e., a “healthy” weight range), and 38.7% reported mostly B’s for grades. Over half (53.4%) of the sample identified as White, non-Hispanic, 10.1% identified as Black or African American, 26.8% identified as Hispanic/Latino, 4.8% identified as Asian, and 4.8% identified as either non-Hispanic multi-racial or other race/ethnicity. Over one third (35.5%) reported engaging in moderate (3–5 days) muscle-building exercise. Nearly one in five (19.8%) participants reported general weapon carrying, 3.3% reported weapon carrying at school, 6.5% reported gun carrying, 28.0% reported general physical fighting, and 10.7% reported physical fighting at school. See Table 1 for a full description of the sample.

**Table 1.** Characteristics of U.S. High School Boys from the 2019 Youth Risk Behavior Survey (*N* = 4120).

	%
Age	
≤14 years	11.3
15 years	25.2
16 years	25.6
17 years	23.0
≥18 years	14.9
Race/ethnicity	
White, non-Hispanic	53.4
Black or African American	10.1
Hispanic/Latino^a^	26.8
Asian	4.8
Other^b^	1.1
Non-Hispanic multi-racial	3.7
Sexual orientation	
Heterosexual	92.0
Gay	1.8
Bisexual	3.6
Not sure	3.0
BMI percentile	
<5th percentile (“underweight”)	3.2
5th ≤ to <85th percentile (“healthy weight”)	56.0
85th ≤ to <95th percentile (“overweight”)	12.9
≥95th percentile (“obese”)	27.9
Grades, past 12 months	
Mostly A’s	33.3
Mostly B’s	38.7
Mostly C’s	18.5
Mostly D’s	4.5
Mostly F’s	1.6
None of these/don’t know	3.4
Bullied at school, past 12 months	14.8
Bullied electronically, past 12 months	10.6
Any team sports participation, past 12 months	60.9
Any days of not attending school due to feeling unsafe, past 30 days	6.3
Any experience of being threatened or injured with a weapon at school, past 12 months	8.1
Muscle-building exercise engagement, past 7 days	
No engagement (0 days)	23.7
Low engagement (1–2 days)	17.4
Moderate engagement (3–5 days)	35.5
High engagement (6–7 days)	23.5
Weapon carrying, past 30 days^c^	19.8
Weapon carrying at school, past 30 days^c^	3.3
Gun carrying, past 12 months	6.5
Physical fighting, past 12 months	28.0
Physical fighting at school, past 12 months	10.7

*Note.* Analyses included preconstructed nationally representative sample weighting. BMI = Body mass index.

^a^Hispanic/Latino included multi-racial Hispanic/Latino.

^b^Other race/ethnicity included American Indian, Alaskan Native, Native Hawaiian, or other Pacific Islander.

^c^Weapon includes gun, knife, or club.

**Figure 1.**
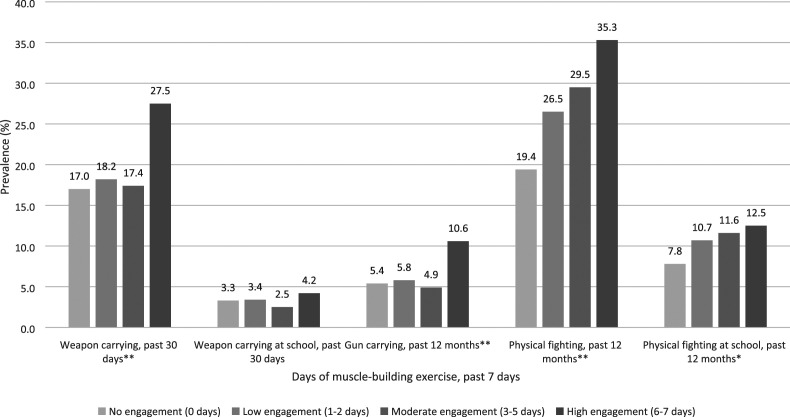
Unadjusted Prevalence of Weapon Carrying and Physical Fighting by Muscle-Building Exercise among U.S. High School Boys from the 2019 Youth Risk Behavior Survey (*N* = 4120) Differences between groups determined by the adjusted *F*, a variant of the second-order Rao-Scott adjusted χ^2^. *p < 0.05 **p < 0.001.

Significant differences in prevalence of weapon carrying and physical fighting emerged in unadjusted analyses. Prevalence of general weapon carrying (27.5%), gun carrying (10.6%), general physical fighting (35.3%), and physical fighting at school (12.5%) were significantly higher among participants who reported high engagement (6–7 days) of muscle-building exercise compared to those who reported no engagement (0 days; Figure 1).

Multiple logistic regression analyses revealed several significant associations between engagement in muscle-building exercise and weapon carrying and physical fighting (Table 2). Compared to no engagement (0 days), participants who reported high engagement (6–7 days) in muscle-building exercise had higher odds of general weapon carrying (adjusted odds ratio [AOR] 2.18, 95% confidence interval [CI] 1.54–3.07), gun carrying (AOR 2.12, 95% CI 1.23–3.64), and general physical fighting (AOR 2.07, 95% CI 1.53–2.79). Similarly, compared to no engagement (0 days), participants who reported moderate engagement (3–5 days) in muscle-building exercise had higher odds of general physical fighting (AOR 1.65, 95% CI 1.25–2.18) and physical fighting at school (AOR 1.63, 95% CI 1.10–2.43).

**Table 2.** Associations between Muscle-Building Exercise and Weapon Carrying and Physical Fighting among U.S. High School Boys from the 2019 Youth Risk Behavior Survey (*N* = 4120).

	Weapon carrying, past 30 days^a^	Weapon carrying at school, past 30 days^a^	Gun carrying, past 12 months	Physical fighting, past 12 months	Physical fighting at School, past 12 months
Muscle-building exercise engagement, past 7 days	AOR^b^ (95% CI)	AOR^b^ (95% CI)	AOR^b^ (95% CI)	AOR^b^ (95% CI)	AOR^b^ (95% CI)
No engagement (0 days)	Ref	Ref	Ref	Ref	Ref
Low engagement (1–2 days)	1.17 (0.81–1.67)	1.28 (0.55–2.99)	1.02 (0.56–1.84)	1.29 (0.92–1.80)	1.27 (0.79–2.04)
Moderate engagement (3–5 days)	11.14 (0.83–1.57)	1.06 (0.49–2.32)	1.05 (0.63–1.75)	1.65 (1.25–2.18)***	1.63 (1.10–2.43)*
High engagement (6–7 days)	2.18 (1.54–3.07)***	1.75 (0.73–4.18)	2.12 (1.23–3.64)**	2.07 (1.53–2.79)***	1.44 (0.94–2.22)

*Note.* Analyses included preconstructed nationally representative sample weighting.

**Boldface** indicates statistical significance (*p* < 0.05). *p < 0.05, **p < 0.01, **p < 0.001.

AOR = Adjusted odds ratio; CI = Confidence interval.

^a^Weapon includes gun, knife, or club.

^b^Adjusted for age, race/ethnicity, sexual orientation, body mass index percentile, grades, bullying victimization (at school and electronically), any team sports participation, any days of not attending school due to feeling unsafe, and any experience of being threatened or injured with a weapon at school.

In sensitivity analyses further adjusting for number of days of at least 60 minutes of physical activity in the past 7 days, results were the same with slightly attenuated effect sizes. Additionally, we assessed whether team sports participation moderated the relationships between muscle-building exercise engagement and weapon carrying and physical fighting, and there were no significant interactions in these analyses.

## Discussion

The results from this study confirm the hypothesis that high school boys who engage in a greater number of days of muscle-building exercise, that is, in excess of the recommended guidance (Stricker et al., 2020), have higher odds of also reporting both weapon carrying and physical fighting. Specifically, those who reported high engagement in muscle-building exercise, compared to no engagement, had over two-fold higher odds of reporting general weapon carrying, gun carrying, and general physical fighting. Further, those who reported moderate engagement in muscle-building exercise, compared to no engagement, also had higher odds of both general physical fighting and physical fighting at school. These findings are particularly salient as they are independent of several potential confounding factors, including multiple forms of bullying victimization, avoiding school due to lack of safety, and being threatened or injured at school with a weapon. Presumably, outside the context of muscle-building exercise, these factors would increase one’s likelihood of carrying a weapon and/or engaging in physical fighting for self-protection (Ganson & Nagata, 2020; Pham et al., 2017; Shetgiri et al., 2015; Van Geel et al., 2014). Additionally, these findings are independent of team sports participation, which would presumably account for the high engagement in muscle-building exercise (i.e., for sports-related purposes) via practices and training (Nagata et al., 2019). Lastly, in sensitively analyses further adjusting for engagement in general physical activity, the findings remained statistically significant, though the effect sizes slightly attenuated, further indicating the independence of muscle-building exercise as an associated behavior with weapon carrying and physical fighting.

Adolescence is a critical time period for psychological and social development, as well as health behavior integration, that has significant impacts on future health and well-being (Patton et al., 2016; Sawyer et al., 2012). While engagement in muscle-building exercise is not inherently problematic, and can in fact be quite beneficial to overall physical health when done in accordance with public health guidance (Stricker et al., 2020; U.S. Department of Health and Human Services, 2018), the findings from this study underscore a potential problematic pattern of behavior. That is, the high engagement in one behavior (i.e., muscle-building exercise) is associated with multiple forms of problematic social behaviors (i.e., weapon carrying and physical fighting). Importantly, even those who reported moderate engagement in muscle-building exercise had greater odds of physical fighting in and outside of school, underscoring that these problematic behavioral patterns may not only occur among boys who engage in high muscle-building exercise. Overall, these findings may be contrary to the assumption that high engagement in physical activity, muscle-building exercise in this case, among adolescent boys is absent of other risk behaviors.

While the YRBS does not include a gender norms variable, thus precluding our ability to investigate the impacts of gender conformity to the findings, prior work may provide context for our findings. For example, adolescent boys are uniquely impacted by gender norm socialization processes that emphasize physical toughness and strength (Amin et al., 2018; Kågesten et al., 2016). Indeed, research has shown that greater gender conformity among boys, that is greater adherence to masculine norms, is associated with involvement in muscularity-oriented behaviors, such as muscle-building exercise (Calzo et al., 2016; Nagata, Domingue, et al., 2020). Relatedly, attempts by boys to display their adherence to masculine norms may occur through behavior such as weapon carrying and physical fighting (Amin et al., 2018; Fleming & Agnew-Brune, 2015). Of course, many adolescent boys are not impacted or influenced by prescribed norms of masculinity and the muscular body ideal; however, the high engagement in muscle-building exercise found in this study (i.e., more than 50% of the sample reported three or more days) may indicate the pervasiveness of these sociocultural norms. A key area for future research would be to determine whether gender norm variables (i.e., hegemonic masculinity) mediates the association of muscle-building exercise on weapon carrying and physical fighting. In addition, future qualitative research would be beneficial to uncover the motivations behind overlapping behaviors of muscle-building, weapon carrying, and fighting among adolescent boys.

The findings from this study should also be contextualized in part by factors related to racial/ethnic identity and sexual orientation. Prior research has shown that involvement in muscularity-oriented behaviors, such as weight lifting, may be more common among Black and Hispanic males compared White males (Nagata, Ganson, et al., 2020; Ricciardelli et al., 2007), as well as sexual minority (i.e., non-heterosexual) males (Calzo et al., 2013). While research on physical fighting has shown differences across races and ethnicities, including higher prevalence among African-American and Hispanic youth than White youth (Salas-Wright et al., 2017), weapon carrying occurs similarly across races and ethnicities (Jewett et al., 2021; Muula et al., 2008; Vaughn et al., 2012). Thus, the adjustment of both demographic identifiers in our analyses shows that the associations found in this study are independent of these factors, which may increase propensity of engage in these behaviors. This further emphasizes the need for prevention and intervention programming that is tailored to all adolescent boys versus one particular demographic group, which may also address issues of health disparities across multiple marginalized groups.

With this context in mind, and given that adolescence is a critical time for gender socialization and the development of interpersonal patterns of behavior and risk taking that may track into adulthood, the findings from this study have important implications for future research and prevention and intervention efforts. First, future research is needed to continue to describe the social and psychological catalysts and facilitators of engagement in muscle-building exercise (Ganson & Rodgers, 2022). Additionally, future research, particularly qualitative data, that clarifies the nuances and complexities of the relationship between muscle-building exercise and weapon carrying and physical fighting. This, and other research using purposively designed surveys, can explore the facilitators and barriers of weapon carrying and physical fighting related to team and organized sports involvement, coaches and athletic trainers, community recreation programming and centers, and the educational environment. Such research will provide greater context to the findings from this study and strengthen the following proposed prevention and intervention efforts. Interventions, including short films and activities/exercises, can be used in schools and sports clubs to address gender equity and violence among adolescent boys (Jewkes et al., 2015), which may be implemented to reduce engagement in risk behaviors, such as weapon carrying and physical fighting. Healthcare practitioners and school professionals should consider assessing for and monitoring risk behaviors, such as weapon carrying and physical fighting, and high engagement in muscle-building exercise among adolescent boys. School policies should be adapted to ensure that appropriate education and monitoring of muscle-building exercise occurs among adolescent boys to ensure the proper engagement in such exercise. This may include coaches and athletic trainers assessing their contribution to promoting high engagement in muscle-building exercise among adolescent boys. In fact, the AAP recommends the involvement of teachers, coaches, and instructors to ensure a safe training environment and the use of developmentally appropriate teaching strategies (Stricker et al., 2020). Community recreation centers and gyms can provide information and educational sessions to members on the potential adverse social correlates of high engagement in muscle-building exercise. Additionally, policies at community recreation centers and gyms can be altered to ensure that adolescent boys complete specific training and education sessions with a personal trainer to ensure adequate knowledge and skills related to muscle-building exercise. However, in order for such interventions to be effective, broader systemic and sociocultural change may be needed to reduce the emphasis on the muscular body ideal for adolescent boys. Public health professionals should consider the correlates of high engagement in muscle-building when promoting physical activity, such as muscle-building exercise, to ensure the adaptive engagement in this form of exercise, as well as consider high engagement in this behavior as a potential correlate of weapon carrying and physical violence among adolescent boys.

### Limitations

Despite the important and novel findings of this study, several limitations should be noted. The YRBS uses a cross-sectional study design, which limits the ability to draw causal inferences from the data. Future research should aim to assess the questions posed in this study with data that use a longitudinal cohort study to identify the prospective relationship between these behaviors. All items in the YRBS are self-reported, which may be increase social desirability and reporting biases. Additionally, the YRBS does not include specific items and measures that could be used to assess why participants engage in muscle-building exercise, weapon carrying, and physical fighting, which should be implemented in future research. Lastly, there is the potential for unmeasured confounders (e.g., socioeconomic status) that influence the associations between the variables under study. Strengths of this study include the large, diverse, and nationally representative sample of U.S. adolescent high school boys.

## Conclusion

In a large and nationally representative sample of adolescent boys, high engagement in muscle-building exercise was significantly associated with general weapon carrying, gun carrying, and general physical fighting, while moderate engagement in muscle-building exercise was significantly associated with physical fighting on and off school grounds. These findings underscore a potentially problematic behavioral pattern associated with high and moderate engagement in such exercise. Prevention and intervention efforts among health care professionals, school personnel, and athletics professionals should ensure that adolescent boys properly engage in muscle-building exercise in ways that are not harmful, via thorough training, education, and with oversight, to potentially reduce associated risk behaviors.
